# Modeling Learning Patterns to Predict Placebo Analgesic Effects in Healthy and Chronic Orofacial Pain Participants

**DOI:** 10.3389/fpsyt.2020.00039

**Published:** 2020-02-12

**Authors:** Yang Wang, Christina Tricou, Nandini Raghuraman, Titilola Akintola, Nathaniel R. Haycock, Maxie Blasini, Jane Phillips, Shijun Zhu, Luana Colloca

**Affiliations:** ^1^Department of Pain and Translational Symptom Science, School of Nursing, University of Maryland, Baltimore, MD, United States; ^2^Center to Advance Chronic Pain Research, University of Maryland, Baltimore, MD, United States; ^3^Department of Neural and Pain Sciences, School of Dentistry, University of Maryland, Baltimore, MD, United States; ^4^Department of Organizational Systems and Adult Health, School of Nursing, University of Maryland, Baltimore, MD, United States; ^5^Departments of Anesthesiology and Psychiatry, School of Medicine, University of Maryland, Baltimore, MD, United States

**Keywords:** conditioning, expectation, latent class analysis, pain, temporomandibular disorder

## Abstract

Successfully predicting the susceptibility of individuals to placebo analgesics will aid in developing more effective pain medication and therapies, as well as aiding potential future clinical use of placebos. In pursuit of this goal, we analyzed healthy and chronic pain patients' patterns of responsiveness during conditioning rounds and their links to conditioned placebo analgesia and the mediating effect of expectation on those responses. We recruited 579 participants (380 healthy, 199 with temporomandibular disorder [TMD]) to participate in a laboratory placebo experiment. Individual pain sensitivity dictated the temperatures used for high- and low-pain stimuli, paired with red or green screens, respectively, and participants were told there would be an analgesic intervention paired with the green screens. Over two conditioning sessions and one testing session, participants rated the painfulness of each stimulus on a visual analogue scale from 0 to 100. During the testing phase, the same temperature was used for both red and green screens to assess responses to the placebo effect, which was defined as the difference between the average of the high-pain-cue stimuli and low-pain-cue stimuli. Delta scores, defined as each low-pain rating subtracted from its corresponding high-pain rating, served as a means of modeling patterns of conditioning strength and placebo responsiveness. Latent class analysis (LCA) was then conducted to classify the participants based on the trajectories of the delta values during the conditioning rounds. Classes characterized by persistently greater or increasing delta scores during conditioning displayed greater placebo analgesia during testing than those with persistently lower or decreasing delta scores. Furthermore, the identified groups' expectation of pain relief acted as a mediator for individual placebo analgesic effects. This study is the first to use LCA to discern the relationship between patterns of learning and the resultant placebo analgesia in chronic pain patients. In clinical settings, this knowledge can be used to enhance clinical pain outcomes, as chronic pain patients with greater prior experiences of pain reduction may benefit more from placebo analgesia.

## Introduction

Placebo effects represent a phenomenon that encompasses psychological, biological, and interpersonal aspects of human physiology and behavior (1). A variety of frameworks, theories, and concepts have been postulated in an attempt to understand how placebo effects are elicited, formed, and maintained over time (2). Placebo effects appear to be complex in nature, highly flexible across contexts, and dynamic over time with their ability to influence symptoms and health outcomes. The high complexity of placebo effects, and the influence of subconscious processes, makes it unlikely that a single mechanism leads to the formation of placebo effects. However, expectations and placebo effects that influence and modify a patient's perception of symptoms may respond to computational rules and predictive models. Büchel et al. postulated the idea that the complex experience of pain is based on the actions of predictive coding (3). The brain is not merely a decoder of signs and signals from the periphery (e.g. nociceptive stimuli), but rather an elegant machine that makes inferences based on prior experiences and anticipatory cues, or expectations (3). Wiech (4) suggested that the experience of pain is an *inferential process* in which prior information and self-healing experiences are integrated to create anticipations of future events by forming a sort of “template” about future painful (and nonpainful) events, thus providing critical elements about how to interpret the ongoing inputs (4). Thus, humans are likely to interpret their experiences based, at least in part, on their own expectations rather than on the experiences themselves (4). As such, expectations are likely to bias perception of symptoms (e.g. pain experience) and signals (e.g. nociceptive stimuli) through brain activation in areas that process and interpret somatosensory input. According to Wiech, when expectations are too “far-fetched,” then a modification of expectations occurs, making pain perception modulation an active and dynamic process that is enabled and primarily modified by learning processes and prior experiences (4).

In this context of pain signaling, a Bayesian computational model based on predictive coding could account for variability in placebo responsiveness (3). Anchisi and Zanon (5) built a Bayesian decision model (fBD) which indicated that placebo effects result from the integration of nociceptive stimuli with past experience (e.g. *via* conditioning), incoming sensorial information (e.g. nociceptive stimuli), and context (e.g. anticipatory cues) (5). In this study, we expanded upon these theories, using the latent class analysis (LCA) approach (6) to determine how learning patterns during conditioning can affect the formation of placebo analgesia. Additionally, we determined how self-reported expectations of pain relief are associated with and mediate placebo analgesia.

## Materials and Methods

Five hundred seventy-nine participants volunteered for this study, of which 380 were healthy participants and 199 were patients suffering from temporomandibular disorder (TMD), **Table 1**. All participants gave written consent to participate in this study and the internal review board of the University of Maryland, Baltimore approved the study (Prot. HP-00068315). Since deceptive information was used during the procedure, healthy participants were debriefed at the end of their experimental round using a study exit form that detailed the nature and the involvement of deception. They were offered the chance to withdraw their data from the study but none did.

**Table 1:** Demographic information for TMD (n = 199) and healthy controls (HC, n=380).

	HC	TMD
**N**	380	199
**Sex**		
Male	150 (39.5%)	52 (26.1%)
Female	230 (60.5%)	147 (73.9%)
**Age**	29.1±10.1	40.6±13.8
**Race**		
American Indian or Alaska Native	2 (0.5%)	0 (0%)
Asian	94 (24.7%)	19 (10%)
Black or African American	88 (23.2%)	64 (32.2%)
White	178 (46.8%)	103 (51.8%)
Mixed Race	18 (4.8%)	12 (6%)
**Household Income**		
$0–$19,999	99 (26.1%)	47 (23.6%)
$20,000–$39,999	57 (15.0%)	42 (21.1%)
$40,000–$59,999	65 (17.1%)	32 (16.1%)
$60,000–$79,999	41 (10.8%)	22 (11.1%)
$80,000–$99,999	35 (9.2%)	13 (6.5%)
$100,000–$149,999	43 (11.3%)	24 (12.1%)
$150,000 or higher	38 (10.5%)	18 (9.5%)
**Educational Attainment**		
Did not complete high school	0 (0%)	0 (0%)
Completed high school	16 (4.2%)	31 (15.6%)
Some college	86 (22.6%)	49 (24.6%)
College graduate	175 (46.1%)	62 (31.2%)
Professional or Postgraduate level	103 (27.1%)	57 (28.6%)

### Eligibility Criteria

All participants were within the ages of 18–65 years and were pre-screened over the phone to determine their eligibility as either a healthy or TMD participant. Participants over 65 years of age were excluded because pain thresholds increase and TMD dysfunctions steadily decrease in prevalence and severity with older age (7, 8).

#### Healthy Participants

Three hundred eighty volunteers were deemed eligible and enrolled as healthy participants based on an in-person screening by trained research personnel. Inclusion was based on their age and ability to speak and understand English. Participants were excluded based on the following criteria: presence of pain disorders; presence of degenerative neuromuscular, cardiovascular, neurological, kidney, or liver disease; pulmonary abnormalities; cancer within the past three years; any uncorrected impaired hearing; color-blindness; and pregnancy or breast-feeding. Participants with a family history of schizophrenia, bipolar disorders, and other psychoses were also excluded, as were those with any severe psychiatric condition leading to hospitalization in the last three years. Lifetime dependence on, or abuse within the prior year of, alcohol or recreational drugs was also an exclusion criteria. Volunteers identified as healthy participants underwent an in-person by trained research personnel who verified the screening results to ensure eligibility. In addition to the criteria listed above, healthy participants were also excluded if they suffered from any chronic pain condition or had a personal history of psychosis.

#### TMD Participants

One hundred ninety-nine volunteers were enrolled as TMD participants. Inclusion criteria for TMD participants were met by those who reported a minimum of 3 months of pain in the jaw, temple, or ear area on either side prior to examination. Those identified as potential TMD patients received an in-person clinical examination by a dental hygienist with expertise in orofacial pain at the Brotman Facial Pain Clinic at the University of Maryland, School of Dentistry. TMD research classifications were confirmed according to the Axis I Diagnostic Criteria for Temporomandibular Disorders (DC/TMD) (9, 10). Axis II instruments were completed and grading of instruments was performed in accordance with the DC/TMD Scoring Manual for Self-Report Instruments (11). Participants were excluded based on the following criteria: presence of cervical pain (following stenosis or radiculopathy); presence of degenerative neuromuscular, cardiovascular, neurological, kidney, or liver disease; pulmonary abnormalities; diffuse cancer within the past three years; any uncorrected impaired hearing; color-blindness; and pregnancy or breast-feeding. Participants with any severe psychiatric condition leading to hospitalization in the last three years, lifetime dependence on alcohol or recreational drugs, or abuse of either within the prior year were also excluded.

### Experimental Procedures

The experiment took place within the Clinical Suites at the University of Maryland Baltimore, School of Nursing and consisted of a single session. The study procedures were described in detail during the consent process, and participants provided written informed consent. Vital signs, including blood pressure, heart rate, height, weight, and body mass index (BMI), were recorded for monitoring purposes only.

### Heat Pain Stimulation

Painful thermal heat stimuli were applied to the dominant forearm and delivered using an ATS 30×30 thermode (PATHWAY System, Medoc, Ramat Yishai, Israel). The participants performed a pain sensitivity assessment using the limits paradigm (12) and reported their pain intensity (ranging from 0 = no pain to 100 = maximum tolerable pain) verbally to the experimenter. The pain sensitivity assessment allowed tailoring of the maximum, moderate, and minimum levels of painful stimulations to each participant, which were then used for the placebo manipulation during the conditioning and testing phases. The participants were reminded about the experimental tasks upon completion of the pain sensitivity assessment. They were informed that they would be receiving both electrical (actual a sham electrode) and heat-pain stimulation while viewing two colored screens, namely red and green. The sham electrode was attached above the thermode on the forearm, and they were informed that the electrode would stimulate their nerves at an imperceptible “subthreshold level” to reduce their pain. They were informed that the electrode would only be active when they viewed a green screen, and not red. The participants were trained to use a script-based rating device (Celeritas Fiber Optics Response System, Psychology Software Tools Inc, Sharpsburg, PA, USA) to rate their pain intensity after every trial using the visual analog scale (VAS) ranging from 0 = no pain to 100 = maximum tolerable pain.

### Placebo Manipulation

**Figure 1:**
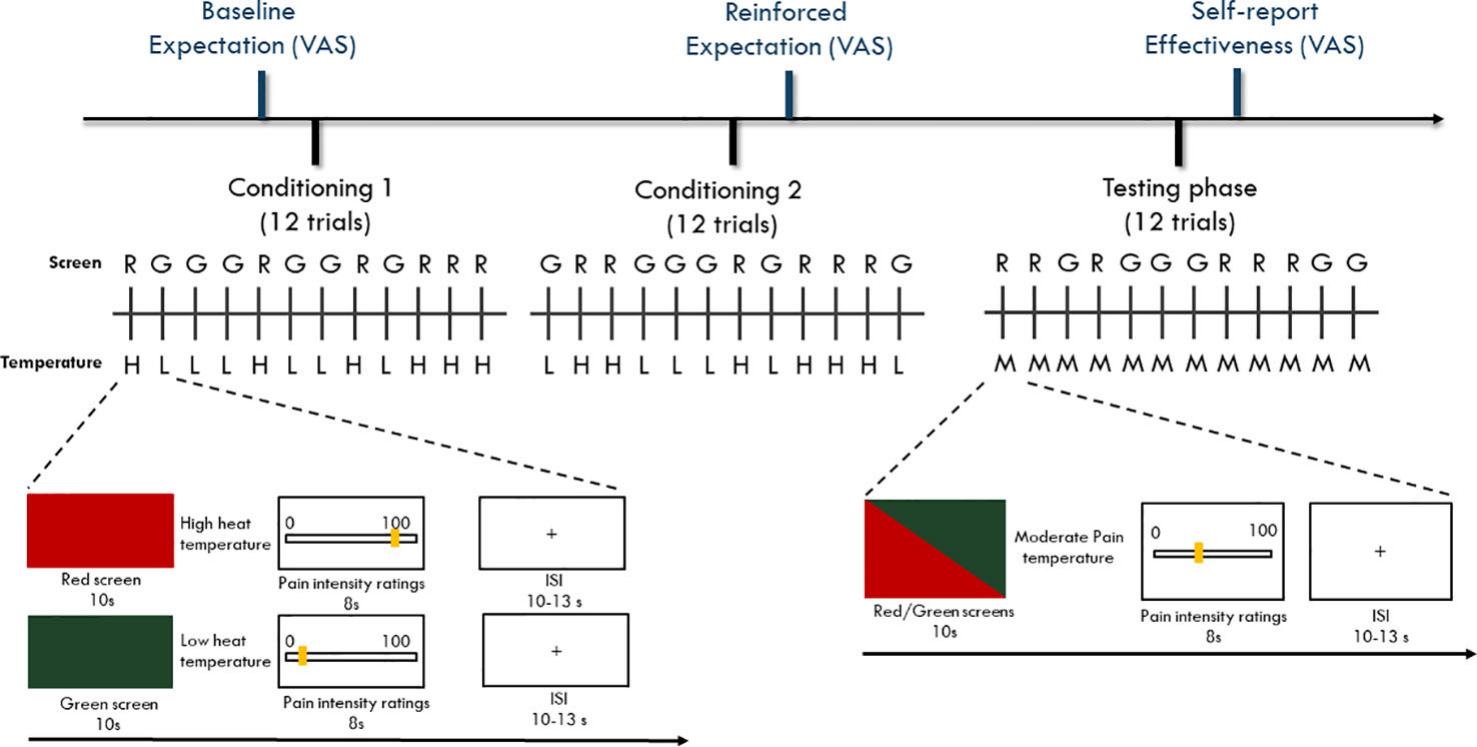
Timeline of the experiment paradigm. Participants went through two sessions of conditioning phase and one session of testing phase with each session containing 12 trials. During the conditioning phase, a red screen was paired with a high heat painful temperature while a green screen was paired with a low heat painful temperature. During the testing phase, both red and green screens were paired with moderate heat painful temperature. For both conditioning and testing phases, the colored screens and the heat painful stimulations were presented for 10 s. After delivery of the heat painful stimulations, a VAS scale with 0 = not painful at all to 100 = maximal tolerable pain was provided (8 s) to assess participants' pain experience ratings. The inter-stimuli-interval (ISI) was set randomly between 10 s to 13 s was presented. For both conditioning and testing phase, red and green trials were randomly displayed using one of the four pre-programmed sequences that are randomly designed.

A well-established conditioning paradigm (13) with two conditioning phases and one testing phase was employed as a placebo manipulation. Each of the two conditioning phases and the testing phase contained 12 heat pain stimulations, of which six stimulations were associated with red screens and six with green screens. The participants were randomized to one of the four pseudorandom sequences of screen color to control for potential sequence effects. The experimenter used the three levels of temperature (accounting for maximum tolerable pain, minimum pain, and moderate pain) from the participant's pain sensitivity assessment. The temperature for the moderate pain level was usually one degree lower than the temperature of the maximum pain level. During the conditioning phases, the temperature for maximum pain was delivered with the red screens, and the temperature for minimum pain was delivered with green screens. During the testing phase, the temperature for moderate pain was delivered with both the red and green screens. After each stimulation, the participants rated their pain intensity using the VAS (**Figure 1**). The difference between the means of the red and green screen ratings during the testing phase was calculated to determine the magnitude of placebo response.

### Expectations Assessments

A 0–100 mm VAS scale was used to assess the participants' self-reported expectation with the question: “How much do you think this procedure will reduce your pain?” Before beginning of the conditioning phase, participants rated their baseline pain relief expectations about the anticipated effectiveness of the intervention. Immediately after the conditioning phase, participants rated their “reinforced expectations” by asking “How much do you think this procedure will reduce your pain?” Finally, after the testing phase, participants rated again their perceived effectiveness of the intervention.

### Statistical Analysis

To examine whether the conditioning procedure induced placebo analgesia, we adopted a repeated measures ANOVA to analyze the differences between the VAS ratings for the red and green trials. The mean delta score of red minus green trials in the testing phase was calculated to compare effect size of placebo analgesia in healthy participants and participants with TMD. The Cohen's d was further calculated. To determine the group differences in expectations ratings at each time point, a repeated measures ANOVA was conducted with the time set as the repeated measure (self-reported expectation at baseline vs. after the conditioning phase vs. after the testing phase) and group (TMD vs. healthy participants) as the between-subjects factor. To identify the variables that needed to be controlled in the latent class analysis (LCA) model, a linear regression model was conducted to explore the influences of demographic variables (sex, age, race, marital status, income, and education, see also **Table 1**), warmth thresholds, and pain sensitivity (i.e., temperature used for the testing phase) on placebo analgesia in the overall sample (n = 579). The variables that showed significant influences on placebo analgesia served as control variables in the LCA model.

The aim of the LCA model was to determine the potential classes that shared similar characteristics to the pain rating patterns during the conditioning phase. To determine the potential classes within TMD and healthy participants, the delta scores of red-minus-green pain ratings during the conditioning phase were modeled using Mplus (14). The variables that showed significant influences on placebo analgesia in linear regression served as control variables for the LCA model. As part of the LCA model, a Lo-Mendel-Ruben Likelihood Ratio Test (LMR-LRT), which is an indication of goodness of fit (15, 16), was used to determine which group separation was ideal. Entropy and Bayesian information criteria (BIC) were used to confirm the separation of classes. Entropy was an index of group separation (17), with larger values indicating greater differences among identified classes. The BIC was set as the goodness of fit criteria, with a smaller value indicating a better model fit (16). Specifically, the optimal number of classes was decided by considering the following requirements: 1) The number of classes (n) was selected when the LMR-LRT was significant (p < 0.05) for *n*-class model and was not significant for the next level of classes (i.e., *n+1*-class model) (15, 16); 2) The entropy value was over 0.8 (17); 3) The classes model had the smallest BIC value, and 4) Each identified classes contained more than 15 participants. We employed a non-parametric test Mann-Whitney U test to assess the statistical relevance of these classes on placebo analgesia.

Finally, we performed mediation analyses within TMD and healthy participants, separately, to test the hypothesis that the identified classes associated with placebo analgesia would be fully mediated by the reported reinforced expectation of pain reductions assessed after the conditioning phase. Mediation analyses were conducted using SPSS marco PROCESS developed by Hayes et al. (18, 19) and expectation scores were set as the mediator (M), placebo analgesia as the dependent variable (Y), and the identified classes as the independent variable (X). For testing indirect effects, a bias-corrected bootstrapping method based on resampling of 5,000 times was used. A 95% bootstrapped confidence interval (BCI) is significant if the interval does not contain zero.

The repeated measurements ANOVA, Mann-Whitney U test, regression and mediation analyses were carried out using the SPSS software package (SSPS Inc, Chicago, Illinois, USA, vers.22) and the Mplus software (vers. 8.2, https://www.statmodel.com/index.shtml) was used for the LCA approaches.

According to previous placebo studies (13, 20), we expected to observe medium to large placebo effects induced by the conditioning paradigm. Based on the within-subjects design (with red and green trials set as the within-subjects factor), we performed a power analysis to determine the minimal number of participants. A total N of 129 would be sufficient to have 0.8 statistical power to observe a medium effect size Cohen's f = 0.25 at the alpha level of 0.05. We also determined the optimal sample size for the LCA algorithm. Assuming that pain ratings during conditioning phase would result in a 2-class model, a minimum N of 109 was needed to achieve 0.8 statistical power to detect a medium effect size (Cohen's ω = 0.44) [(21), Table 8]. Thus, the current study with 199 TMD participants and 380 healthy participants allowed us to determine placebo effects, as well as the underlying conditioning strength pattern, with a full power (>0.8).

## Results

### Pain Ratings During the Conditioning Phase

An omnibus ANOVA for repeated measurements was conducted with red and green trials set as the within-subjects dependent variable and group (TMD vs. healthy participants) as the between-subjects variable. The significant main effect of the condition (F_1,577_ = 6633.11, p < 0.001, Cohen's f = 3.39) indicated that, overall, participants rated red screen pain (mean = 69.64, sem = 0.68) as significantly different than green screen pain (mean = 9.67, sem = 0.41, p < 0.001). The significant interaction (F_1,577_ = 20.44, p < 0.001) between the condition (red vs. green trials) and the group indicated that TMD participants showed a smaller red-minus-green difference (mean = 56.64, sem = 1.19) during conditioning phase than healthy participants (mean = 63.30, sem = 0.86, p < 0.001; Cohen's d = 0.38). Moreover, the significant main effect of the group (F_1,577_ = 9.87, p = 0.002) suggested that, during the conditioning phase, TMD participants reported significantly lower overall pain intensities (mean = 38.65, sem = 0.68) than healthy participants (mean = 40.98, sem = 0.49). A separate analysis for controls and cases was also included and we observed a significant difference in pain ratings between red and green stimulations in both TMD (F_1,198_ = 2182.82, p < 0.001; Red: mean = 66.66, sem = 1.08; Green: mean = 10.02, sem = 0.68; Cohen's f = 3.32) and healthy participants (F_1,379_ = 5468.46, p < 0.001; Red: mean = 72.62, sem = 0.80; Green: mean = 9.32, sem = 0.47; Cohen's f = 3.79).

### Expectation Changes Across Time

There was a significant main effect of time on self-reported expectations (baseline vs. after the conditioning phase vs. after the testing phase, F_2,1148_ = 393.16, p < 0.001). *Post-hoc* analyses applying Bonferroni correction indicated that baseline expectations were significantly lower (mean = 43.92, sem = 1.05) than both reinforced post- conditioning expectations (mean = 74.75, sem = 1.06, p < 0.001) and overall expectations after the testing phase (mean = 68.73, sem = 1.15, p < 0.001). As anticipated, reinforced expectations after the conditioning phase were higher than overall expectations after the testing phase (p < 0.001). There were no differences between groups (TMD vs. healthy participants, F_1,574_ = 0.37, p = 0.542), indicating that TMD and healthy participants had comparable expectations at baseline, after the conditioning phase, and after the testing phase.

### Placebo Analgesia Induced by Conditioning Procedure

An omnibus ANOVA for repeated measurements was conducted with red and green trials during the testing phase set as within-subjects dependent variable and group as between-subjects variable. The significant main effect of the condition (F_1,577_ = 631.03, p < 0.001, Cohen's f = 1.05) indicated that, overall, participants displayed placebo analgesia induced by the conditioning procedure with significantly lower pain intensity ratings for green trials (mean = 30.75, sem = 0.87) in comparison with red trials (mean = 49.73, sem = 0.94, Cohen's d = 1.05). There was no significant interaction between the condition and group (F_1,577__=_ 3.29, p = 0.070), suggesting that the placebo analgesia was similar in the TMD (mean = 17.60, sem = 1.22) and healthy participants (mean = 20.35, sem = 0.89). We observed significant placebo analgesia through a separate analyses for TMDs and healthy participants, as revealed by the main effect of the condition (red vs. green) on pain intensity ratings during the testing phase in both TMDs (F_1,198_ = 197.75, p < 0.001; Cohen's f = 1.00) and healthy participants (F_1,379_ = 540.78, p < 0.001; Cohen's f = 1.19). That is, pain ratings for test trials (green) were significantly lower than control trials (red) during the testing phase in both TMDs (Green: mean = 31.17, sem = 1.37; Red: mean = 48.78, sem = 1.45; p < 0.001) and healthy participants (Green: mean = 30.33, sem = 1.04; Red: mean = 50.68, sem = 1.13; p < 0.001).

### Identifying Critical Covariates

The results of linear regression indicated that older age was associated with lesser placebo analgesia (β = −0.20, p < 0.001). Additionally, higher warmth-detection threshold was associated with lesser placebo analgesia (β = −0.10, p = 0.020). Given that age and warmth-detection thresholds had a significant impact on placebo analgesia, those two variables were treated as covariates in the LCA models.

### Latent Class Analysis

**Figure 2:**
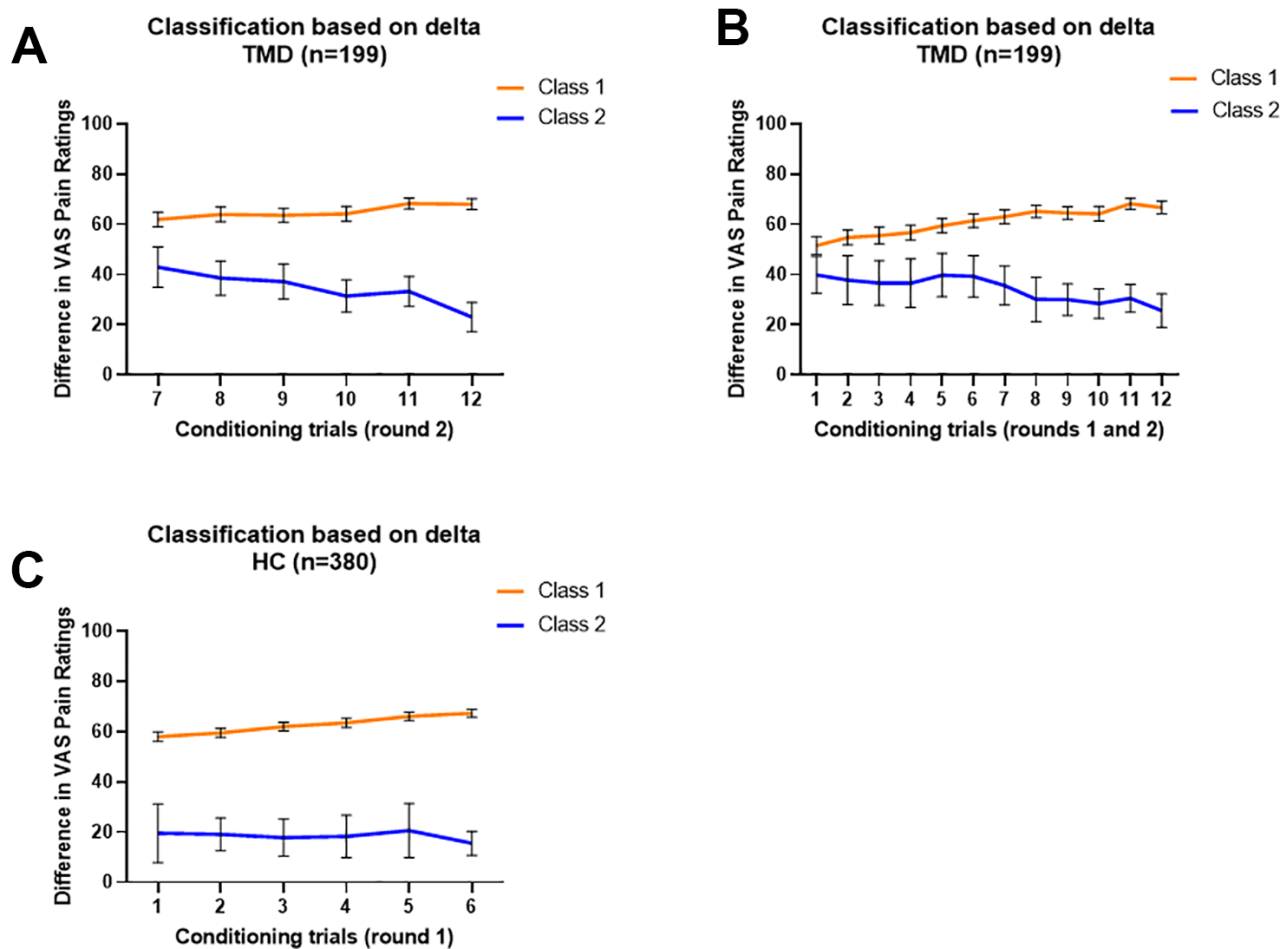
Characterization of classified trajectories. In all models, Class 1 is orange and Class 2 is blue. **(A)** Delta value trajectories of the second conditioning round provided a model of TMD participants in which Class 1 (n = 164) displayed a persistent large delta score and Class 2 (n = 35) displayed a decreasing delta score. **(B)** Delta value trajectories of both conditioning rounds provided a model dividing TMD participants into Class 1 (n = 167), gradually increasing in delta score, and Class 2 (n = 32), gradually decreasing in delta score. **(C)** Delta value trajectories of the first conditioning round provided a model which divides healthy participants into Class 1 (n = 360), displaying increasing but persistently large delta scores, and Class 2 (n = 20), displaying lower and fluctuating delta scores. No significant classes were identified when rounds 1n and 2 were considered together in healthy participants. Data are expressed as delta and error bars show 95% Confidence Interval.

We modeled the trajectory of the effects of learning using the delta scores of red-minus-green pain intensity ratings during the conditioning phase. For TMDs, the LMR-LRT was significant for the 2-Class model according to the delta pain ratings during the second round of conditioning (p = 0.035) with a high entropy value (0.858, **Table 2**). This suggests that placebo conditioning trajectories differed substantially during the second round of the conditioning phase between the two subgroups. The goodness of fit criteria were adequate with BIC = 9839.827 for this model. Class 1, including 164 participants (82.4%), was characterized by persistent large delta scores of pain ratings. On the contrary, Class 2 (35 participants, 17.6%) was characterized by a decreasing delta scores over trials (**Figure 2A**).

**Table 2:** Goodness-of-fit criteria for LCA models within TMD and healthy controls (HC).

Groups	Conditioning Trials Used	# of classes	BIC	Sample-Size Adj. BIC	Entropy	LMR LRT test		# participants in each class
						Test value	p-value	
**TMD**	1-6	2	10,102.429	10,045.404	0.971	1.199	0.1496	4/195
		3	10,093.484	10,026.955	0.887	16.283	0.2353	168/4/27
**TMD**	**7-12**	**2**	**9,896.852**	**9,839.827**	**0.858**	**42.981**	**0.0354**	**164/35**
		3	9,895.995	9,829.466	0.881	15.745	0.2229	158/29/12
**TMD**	**1-12**	**2**	**19,878.389**	**19,802.356**	**0.812**	**24.180**	**0.0499**	**167/32**
		3	19,882.965	19,797.428	0.865	10.635	0.3876	11/27/161

**HC**	**1-6**	**2**	**18,200.348**	**18,143.238**	**0.892**	**30.298**	**0.0205**	**360/20**
		3	18,108.021	18,124.136	0.793	25.948	0.1071	19/150/211
**HC**	7-12	2	17,511.885	177,454.774	0.889	44.747	0.2961	27/353
		3	17,476.142	17,409.513	0.959	50.717	0.0635	353/22/5
**HC**	1-12	2	35,339.347	35,463.199	0.815	37.505	0.1012	335/45
		3	35,340.326	35,254.660	0.834	15.946	0.6207	333/18/29

Additionally, the TMD participants were classified into two classes based on delta scores using the overall conditioning phase (12 trials in total). The LMR-LRT test was significant (p = 0.0499) with adequate entropy value (0.812), suggesting that the two subgroups showed distinct trajectory patterns of delta scores. The goodness of fit criteria were adequate with BIC = 19878.389 for this model. Class 1, including 167 participants (83.9%), was characterized as gradually increasing delta scores over trials. On the contrary, Class 2 (32 participants, 16.1%) was characterized as gradually decreasing delta scores over trials (**Figure 2B**).

For healthy participants, the LCA model for the first round of conditioning resulted in a 2-Class model (LMR-LRT test p = 0.021) with a high entropy value (0.892), suggesting differences in the trajectory patterns of conditioning between the two subgroups. The goodness of fit criteria were adequate with BIC = 18200.348 for this model. Class 1, including 360 participants (94.7%), was characterized as having increasing effects of conditioning over trials, with greater subsequent placebo analgesia. Class 2 (20 participants), on the other hand, was characterized as having persistently lower effects of conditioning, with fluctuating changes in subsequent placebo analgesia over the trials (**Figure 2C**). The remaining LCA models, which did not meet the criteria, are reported in **Table 2**.

### Class Differences in Placebo Analgesia

**Figure 3:**
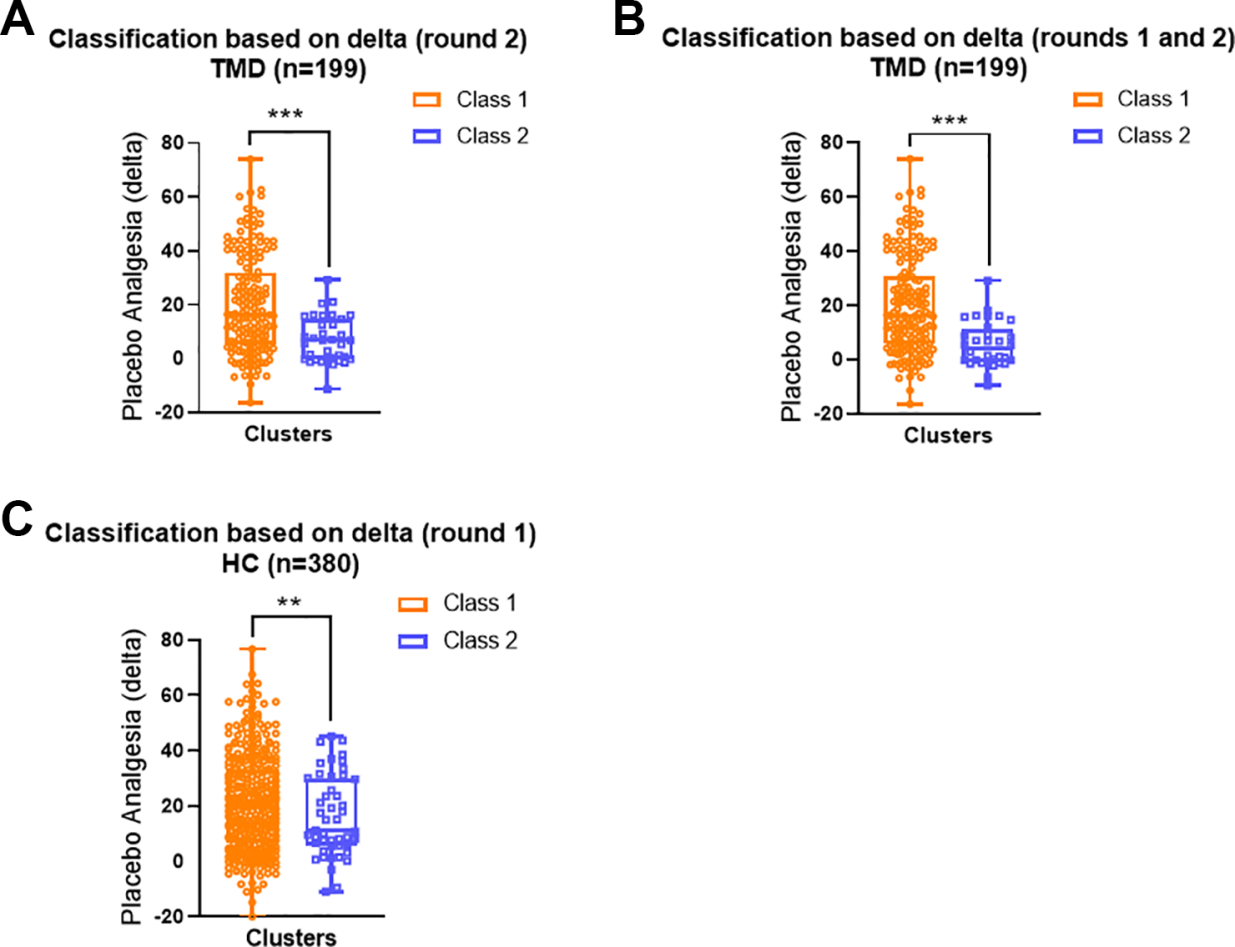
Distribution of individual placebo analgesic scores based on the LCA classes. **(A)** In the TMD participants, Class 1 (the second conditioning round) displayed greater individual placebo effects than Class 2 (Mann-Whitney U = 1650.5, p < 0.001). **(B)** In the TMD participants Class 1 displayed greater individual placebo effects than Class 2 when both conditioning rounds were included (Mann-Whitney U = 1335.5, p < 0.001). **(C)** In the healthy participants, Class 1 displayed greater individual placebo effects than Class 2 when round 1 of conditioning was detected as significant (Mann-Whitney U = 2180.5, p = 0.003). In all models, Class 1 is orange and Class 2 is blue. Data are presented as box-and-whisker plots with median, quartile, and minimum and maximum represented **p < 0.01; ***p < 0.001.

The grouping based on delta scores during the conditioning phase significantly predicted placebo analgesia within both TMD (**Figures 3A, B**) and healthy participants (**Figure 3C**). For TMD participants, those who showed persistently large differences between red and green trials in the second round of conditioning (Class 1) displayed significantly higher placebo analgesia in the testing phase than participants from Class 2, who showed reducing differences between red and green trials during the conditioning phase (Mann-Whitney U = 1650.5, p < 0.001, **Figure 3A**). When classes were identified based on delta scores across the whole conditioning phase (12 trials), the results indicated that TMD participants who showed increasing differences between red and green pain ratings over trials also had greater placebo analgesia(Mann-Whitney U = 1335.5, p < 0.001, **Figure 3B**). These results indicated that larger and increasing delta scores during the conditioning phase were associated with greater placebo analgesia within TMD participants.

Similarly, healthy participants who reported more substantial differences between red and green trials during the first round of conditioning (Class 1) displayed significantly larger placebo analgesia compared to those who reported consistently small differences between red and green trials during the conditioning phase (Class 2, Mann-Whitney U = 2180.5, p = 0.003, **Figure 2C**).

### Class Differences in Expectations

We determined the class differences in self-reported expectations (baseline vs. after conditioning phase vs. after testing phase) within TMD and healthy participants, separately. For TMD participants, rwo classes were identified based on session 2 of the conditioning phase (trial 7 to trial 12). Class 1 was characterized by greater conditioning strength while class 2 was characterized by smaller conditioning strength. Those two classes did not differ in baseline expectations (Mann-Whitney U = 2857.0, p = 0.966) or self-reported effectiveness after the testing phase (Mann-Whitney U = 2662.5, p = 0.501). However, class 1 displayed significantly greater reinforced expectations after the conditioning phase (mean rank = 106.44) in comparison with class 2 (mean rank = 69.81, Mann-Whitney U = 1813.5, p = 0.001). TMD participants were also classified into two classes based on overall conditioning phase (trial 1 to trial 12). Class 1 was characterized by greater overall conditioning strength while class 2 was characterized by less overall conditioning strength. Class 1 and class 2 did not show any differences in baseline expectations ratings (Mann-Whitney U = 2365.50, p = 0.296). However, those TMD participants with greater overall conditioning strength (class 1), showed greater reinforced expectations (Mann-Whitney U = 1364.00, p < 0.001) and self-reported effectiveness after the testing phase (Mann-Whitney U = 1976.00, p = 0.019) than those with lower level of conditioning strength (class 2).

In terms of healthy participants, two classes were identified based on session 1 of the conditioning phase (trial 1 to trial 6). Class 1, which was characterized by greater delta scores during first session of the conditioning phase, showed greater reinforced expectations (mean rank = 191.97) in comparison with class 2 characterizing by smaller conditioning strength (mean rank = 132.97, Mann-Whitney U = 2336.50, p = 0.021). Class 1 and class 2 did not show any differences in baseline expectation ratings (Mann-Whitney U = 3415.50, p = 0.711) or self-reported effectiveness after the testing phase (Mann-Whitney U = 3417.50, p = 0.717).

### Mediation Analysis

**Figure 4:**
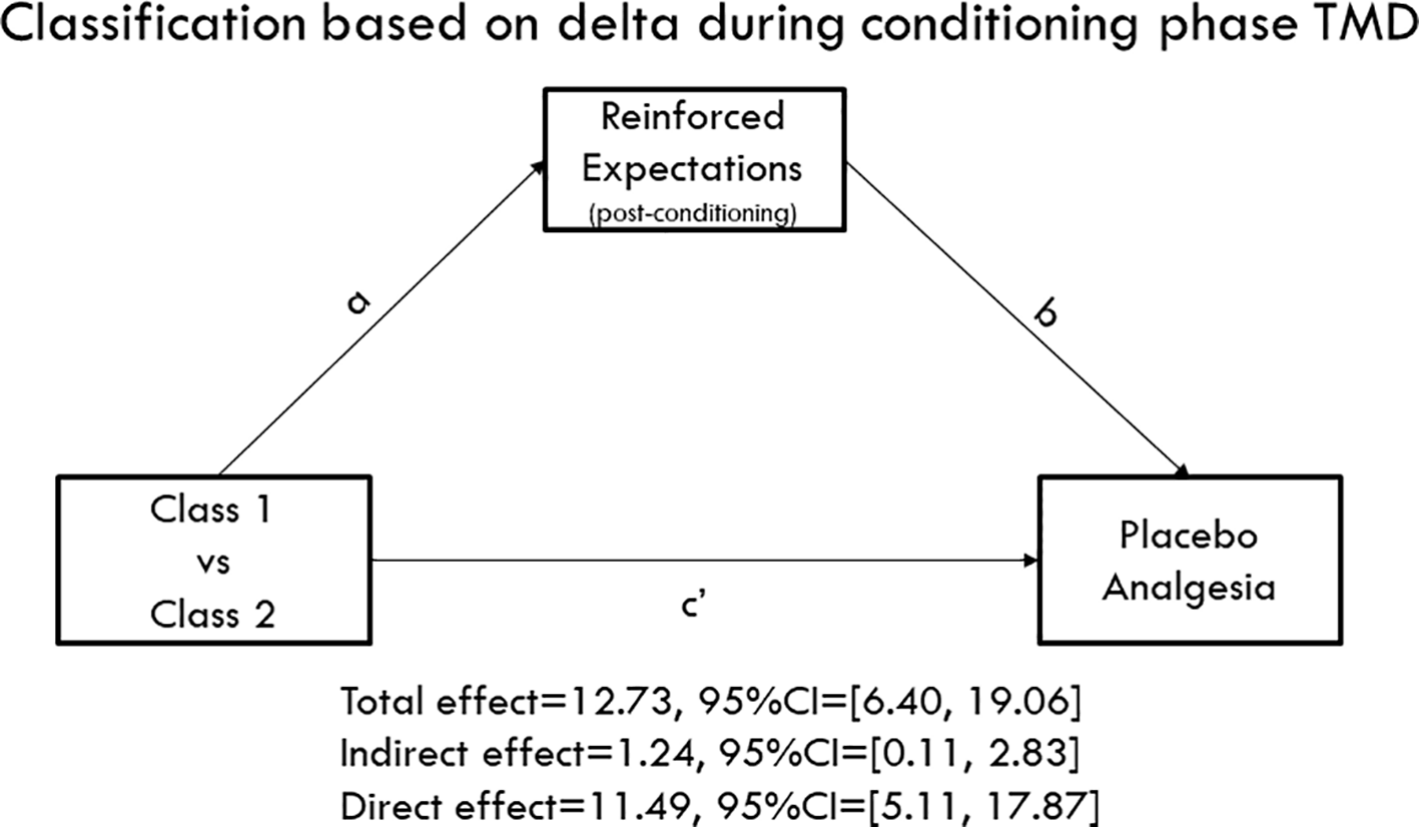
Mediation model. Class 1 displayed persistently larger delta scores of pain ratings during the conditioning phase, while Class 2 showed decreasing delta scores. The mediation model indicated that both the direct effect (c' = 11.49, 95%CI = [5.11, 17.87]) and indirect paths (ab = 1.24, 95%CI = [0.11, 2.83]) were significant, suggesting that conditioning patterns influence the formation of expectations that consequently mediated the association between classes and placebo analgesia during the testing phase.

We tested the hypothesis that the reported reinforced expectations of pain reductions would mediate the association between the identified classes and placebo analgesia observed during the testing phase. Interestingly, when TMD classes were identified based on the second round of conditioning, we found that both the direct effect (c' = 11.49, 95%BCI = [5.11, 17.87]) and indirect effect (ab = 1.24, 95%BCI = [0.11, 2.83]) were significant, suggesting that expectations partially mediated the association between both classes (**Figure 4**). Namely, in comparison to class 2, class 1 was characterized as having larger delta scores in the second round of conditioning and displayed larger placebo analgesia during the testing phase by inducing higher expectations of pain reduction. However, the indirect effect was not significant when the TMD class was identified based on the whole conditioning phase (12 trials) with ab = 1.43, 95%BCI = [−0.08, 3.43], suggesting that the second round conditioning played a more critical role in inducing placebo analgesia. We found that classes of healthy participants were different in expectations levels (Mann-Whitney U = 2513.50, p = 0.023) with class 1 displaying a significantly higher level of pain reduction expectations than class 2. However, the indirect effect was not significant (ab = −0.31, 95%BCI = [−1.45, 0.96]), suggesting that expectations did not mediate the association between the classes and placebo analgesia in healthy volunteers.

## Discussion

This study used an LCA approach to determine how learning patterns during a conditioning phase can affect the formation of placebo analgesia in TMD and healthy participants. It further investigated the relationship between expectations and placebo analgesia. We found that the class of participants with larger perceived differences (delta scores) during conditioning between the maximally painful (red) and minimally painful (green) stimuli exhibited larger placebo effects than those with smaller delta scores. Expectations of pain relief rated after conditioning procedure were larger in those who reported larger differences during testing phase, mediating the subsequent placebo analgesic effects.

LCA is a method to uncover unobserved subgroups where group members share homogeneous characteristics in measured variables (16, 22). This method has been broadly used to explore subtypes of different symptoms, such as eating disorder (23), attention deficit/hyperactivity disorder (24, 25), post-traumatic stress disorder (26, 27), borderline personality disorder (28), and low back pain (29). The advantages of LCA are its flexibility in dealing with both simple and complex data and its rigor in choosing of class criteria (30). Given that prior experience and expectation are critically associated with placebo effects (1), these advantages enabled us to apply this approach to identify learning patterns that could eventually be associated with placebo analgesic effects.

In this study, the LCA-generated models classified both TMD and healthy participants as either showing larger (class 1) or smaller (class 2) delta scores during the conditioning phase. Not surprisingly, the classes with larger delta scores also displayed significantly greater placebo analgesia than those with smaller delta scores. The TMD participants characterized as having larger delta scores in the second round of conditioning displayed greater placebo analgesia during the testing phase by inducing higher expectations of pain reduction than the TMD participants who had smaller delta scores during the conditioning phase. This is in line with previous studies, although to our knowledge our approach is the first one that has looked specifically at learning patterns that drive placebo analgesic effects.

Placebo analgesia describes the beneficial results of a treatment that are due to context, rather than the actions of a drug (1, 3, 31). Previous studies have postulated and demonstrated that prior experiences (pain ratings during a conditioning phase), sensory information (intensity of painful stimuli), and context (cues) all contribute to the formation of placebo analgesia (3–5). Classical conditioning, which forms the expectation of pain relief through reinforcement association, is one of the most effective means of exploring how prior experiences can shape placebo analgesic effects (3), and has been found to induce stronger placebo effects than a mere verbal suggestion procedure [see review, (32)]. We expanded previous findings on conditioned placebo analgesia (13, 33) by showing that distinct learning patterns during a conditioning phase were associated with distinct subsequent placebo analgesic effects. Specifically, participants who had persistently large and/or increasing delta scores during the conditioning phase also displayed greater placebo analgesia than those who showed persistently lower and/or decreasing delta scores during the conditioning phase. This held true in both TMD and healthy participant groups. Indeed, our results highlight the important role of prior experience (i.e., the associational processes during classical conditioning) in shaping placebo analgesia. The results were also meaningful in a clinical context, given that TMD participants who suffer from chronic orofacial pain demonstrated a significant impact of learning patterns on placebo effects. Our findings are also in line with previous clinical studies exploring the association between experiences of treatments and placebo effects (34), where the authors found that previous successful treatments would result in greater placebo effects.

Given that the majority of studies in the area of placebo and pain research have been conducted in healthy, pain-free volunteers, an open question is to what extent we can translate the wealth of knowledge on neurobiological mechanisms of endogenous nociceptive inhibition, or how neural and biological systems interact to block perception of painful stimuli, to populations of pain patients (35). Our findings show that patients suffering from chronic orofacial pain experience placebo effects and therefore may benefit from the activation of descending pain modulation systems and cognitive modulation of expectancy. Our results support findings from studies with pain populations such as chronic irritable bowel syndrome (36–38), idiopathic and neuropathic pain (39–41), low back pain (42, 43), migraine (44), and knee osteoarthritis (45). In addition, these findings align with previous results comparing healthy participants and participants with chronic pain (46, 47).

The significant LCA classes discovered in our study suggest that distinct patterns during conditioning for both TMD and healthy participants induced significant placebo analgesia. Although TMD and healthy participants showed similar overall placebo analgesia (during the testing phase), the two cohorts displayed *different* learning strategies during the conditioning phase. The results of LCA modeling indicate that the TMD patients' learning patterns in the second round of conditioning and healthy participants' learning patterns in the first round of conditioning were associated with the magnitude of their placebo analgesia during the testing phase. The TMD participants were relatively slower in acquiring conditioned pain responses compared to healthy pain-free participants.

Prior experiences not only contribute to the formation of placebo effect per se, but they may also shape pain relief expectations (33). In fact in this study, the link between learning patterns and placebo analgesia was partially mediated by the magnitude of pain-relief expectations. Namely, TMD participants with larger delta scores during the conditioning phase also had higher pain-relief expectations, which in turn induced larger placebo effects. For the healthy participants, too, those who had persistently larger delta scores displayed greater placebo analgesia than those who reported persistently smaller delta scores. In other words, prior analgesic experiences critically and dynamically affected expectations. Although the mediation model determined that self-reported expectations did not significantly mediate individual placebo analgesia, we found that healthy participants with greater delta scores displayed higher pain-reduction expectations than those with smaller delta, indicating a strong influence of learning patterns on the formation of expectations.

The current study has several limitations. First, there was an unequal distribution of participants in our LCA approach, specifically within the identified classes and patterns of responses in the conditioning phase. This unequal distribution may indicate that our model described outlying patterns, which may limit its applicability to a given individual. Moreover, clinical translatability of our modeling approach is hampered because it relies on the conditioning procedure and experimental pain. A clinician treating a single patient may not be able to directly test how that patient responds to conditioning cues and acute experimental pain. Additionally, our paradigm only investigated placebo effects using an acute pain stimulus, and yet, given the high load of pharmacological analgesics consumed by chronic pain patients, the clinical use of placebos is frequently discussed for treatment of chronic pain. Moreover, according to Wiech (4), expectations that are too “far-fetched” may result in updated expectations. The current data only contained expectations rated after the conditioning, which would not allow us to make inferences about the dynamic expectation modulation processes induced by prior experiences. Finally, the exploratory nature of the LCA algorithm used in this study limited the generalization of the present results to a broader population. Future research is required to confirm the underlying learning patterns of chronic pain patients and to determine the associated placebo responsiveness at the individual level, which can help optimizing individualized therapeutic strategies.

Aside from the limitations, the strengths of the current study need to be outlined. First, this is the *first* study exploring placebo analgesia in chronic orofacial pain (1). Second, this is first study to use LCA modeling of a response pattern during the conditioning phase of a well-controlled experimental setting for placebo analgesia. This method enabled us to unveil distinct patterns that were not set *a priori*, and thus the classes we identified emerged naturally. Finally, the current study was the first to demonstrate that TMD participants experience similar conditioned placebo analgesia to healthy pain-free controls. This is critical, given that TMD participants had substantially different prior pain and treatment experiences than healthy controls. Understanding what is similar and what is different in the development of placebo analgesia across the two populations is valuable to successfully developing future treatments.

Although this study provided experimental evidence about how prior experience may influence placebo responsiveness, it still had some clinical implications. First, we found that TMD participants who had suffered from ongoing chronic pain displayed comparable placebo analgesia in comparison with healthy participants, suggesting that chronic pain patients could benefit from placebo procedures as much as healthy populations do. More importantly, it is likely that chronic pain participants who have prior experiences of substantial pain reduction will benefit more from expectancy-induced analgesia in comparison with those who have not had pain relief experiences. In clinical settings, healthcare professionals may need to consider prior therapeutic experiences of patients and expectations of treatment effectiveness when providing treatment plans.

## Conclusion

Placebo analgesia was induced in chronic orofacial pain and pain-free study participants *via* a conditioning procedure, and patterns of their response to placebo during conditioning and testing phases were analyzed. LCA was conducted to classify the participants based on the trajectories of their pain ratings during the conditioning rounds. Participants were grouped into two classes: one characterized by persistently greater differences in their pain ratings during the conditioning rounds and one by persistently lower differences in their pain ratings during the conditioning rounds. Both TMD and pain-free participants in the first class displayed greater placebo analgesia than those in the second class. Furthermore, expectation acted as a mediator for this relationship. This is the first study exploring TMD and LCA in estimating placebo analgesic responsiveness. Modeling therapeutic effects of placebo has large implications for healthcare, specifically in terms of optimizing clinical trial design and even developing personalized therapeutic strategies. Chronic pain patients with greater prior pain relief experiences may respond more to placebo procedures when compared those without previous pain reduction experiences. Healthcare providers should consider prior therapeutic experiences of the patients and assess their expectations of treatment effectiveness when providing pain therapies.

## Data Availability Statement

The raw data supporting the conclusions of this article will be made available by the authors, without undue reservation, to any qualified researcher.

## Ethics Statement

This study was carried out in accordance with the recommendations of the Institutional Review Board (IRB), University of Maryland Baltimore with written informed consent from all subjects. All subjects gave written informed consent in accordance with the Declaration of Helsinki. The protocol was approved by the IRB, University of Maryland Baltimore.

## Author Contributions

LC designed the study and contributed to data analyses. TA, NH, CT, MB, and NR collected the data. JP screened and confirmed the diagnosis of TMD. CT analyzed the data in collaboration with LC and YW. LC, SZ, and YW contributed to the interpretation of the results. LC drafted the manuscript in collaboration with CT, YW, and NR. All authors commented on and approved the final draft.

## Funding

This research is supported by NIDCR (R01 DE025946, LC). The funding agencies have no roles in the study. The views expressed here are the authors' own and do not reflect the position or policy of the National Institutes of Health or any other part of the federal government. We acknowledge the support of the University of Maryland Baltimore, Institute for Clinical & Translational Research (ICTR).

## Conflict of Interest

LC reported having received support for Invited Lectures outside the submitted work.

The remaining authors declare that the research was conducted in the absence of any commercial or financial relationships that could be construed as a potential conflict of interest.

The reviewer KM declared a past co-authorship with one of the authors LC to the handling editor.
